# Multi-mechanical waves against Alzheimer’s disease pathology: a systematic review

**DOI:** 10.1186/s40035-021-00256-z

**Published:** 2021-09-24

**Authors:** Francisca Monteiro, Ioannis Sotiropoulos, Óscar Carvalho, Nuno Sousa, Filipe S. Silva

**Affiliations:** 1 Center for Microelectromechanical Systems (CMEMS), University of Minho, Campus Azurém, 4800-058 Guimarães, Portugal; 2 Life and Health Sciences Research Institute (ICVS), Medical School, University of Minho, Campus Gualtar, 4710-057 Braga, Portugal; 3 ICVS/3B’s - PT Government Associate Laboratory, Braga/Guimarães, Portugal; 4 Institute of Biosciences & Applications NCSR “Demokritos”, Athens, Greece

**Keywords:** Alzheimer’s disease, Auditory stimulation, Mechanical vibrations, Ultrasounds, Whole-body vibration

## Abstract

Alzheimer’s disease (AD) is the most common cause of dementia, affecting approximately 40 million people worldwide. The ineffectiveness of the available pharmacological treatments against AD has fostered researchers to focus on alternative strategies to overcome this challenge. Mechanical vibrations delivered in different stimulation modes have been associated with marked improvements in cognitive and physical performance in both demented and non-demented elderly. Some of the mechanical-based stimulation modalities in efforts are earlier whole-body vibration, transcranial ultrasound stimulation with microbubble injection, and more recently, auditory stimulation. However, there is a huge variety of treatment specifications, and in many cases, conflicting results are reported. In this review, a search on Scopus, PubMed, and Web of Science databases was performed, resulting in 37 papers . These studies suggest that mechanical vibrations delivered through different stimulation modes are effective in attenuating many parameters of AD pathology including functional connectivity and neuronal circuit integrity deficits in the brains of AD patients, as well as in subjects with cognitive decline and non-demented older adults. Despite the evolving preclinical and clinical evidence on these therapeutic modalities, their translation into clinical practice is not consolidated yet. Thus, this comprehensive and critical systematic review aims to address the most important gaps in the reviewed protocols and propose optimal regimens for future clinical application.

**Supplementary Information:**

The online version contains supplementary material available at 10.1186/s40035-021-00256-z.

## Introduction

Alzheimer’s disease (AD) is an age-related neurodegenerative disorder characterized by progressive structural and functional lesions in the brain, which include neuronal atrophy and synaptic loss accompanied by neuronal death, brain network damage, and aberrant network oscillations [1]. These brain lesions are correlated with the accumulation and aggregation of amyloid β (Aβ) peptide and Tau protein [2–4], resulting in extracellular Aβ plaques and intracellular neurofibrillary tangles of hyperphosphorylated Tau protein in the AD brain [5, 6]. This disorder is clinically characterized by evolving memory impairments followed by broad cognitive decline and brain degeneration that ultimately leads to death. AD appears to be a devastating disorder that affects approximately 40 million people worldwide while this number is expected to be triplicated by 2050 [7, 8]. Based on its increasing incidence and high societal impact, the World Health Organization has declared AD and dementia a major priority health issue [8]. However, besides the extensive efforts over years to find a pharmacological treatment for AD, there is no effective therapy that can stop or revert brain pathology as well as cognitive and other behavioral deficits of AD [9].

Recently, alternative therapeutic strategies (e.g. electrical-, magnetic-, optical- and mechanical-based therapies) for AD have been intensively investigated [10–12]. Among the most promising ones, the mechanical wave-based modalities acting at different vibration modes have been associated with improvement of AD neuropathology beyond the reduction of Aβ and Tau accumulation, affecting various pathological parameters involved in AD such as chronic neuroinflammation, synaptic loss, and neuronal atrophy; importantly, these alterations are also accompanied by an attenuation of cognitive and other behavioral deficits. These modalities are non-invasive, low-cost, and easily customized brain stimulation techniques that include whole-body vibration (WBV), transcranial ultrasound stimulation (TUSS), and auditory stimulation (AS). The WBV training consists of passive exercise training delivered by a vibratory platform [13, 14] and has been associated with numerous beneficial effects on neuromuscular function [15–17], vascular diseases [18], and neurological conditions [19, 20] in older adults. During the last five years, a few studies have tested WBV in demented or AD patients, showing promising results [21, 22]. Moreover, the use of TUSS, another mechanical-based modality, in experimental animals has also provided some solid evidence on its beneficial effects on brain plasticity and function as well as  neuronal circuit integrity in young wild-type animals [23–29]. More recent studies support the use of the AS, a sensory-based stimulation strategy delivered by trains of tones, as a potential solution to entrain gamma oscillation in the brains of AD transgenic mice, which has been shown to reduce AD neuropathology and behavioral impairment [30, 31].

These mechanical-based therapeutic modalities are in the early exploration of alternative therapies to attenuateAD pathology and associated behavioral deficits. Although they share the same fundamental energy-transfer carrier (which is the mechanical waves), the stimulation modalities require different application protocols and acoustic/mechanical parameters, and therefore distinct specifications must be considered. In addition, there is a huge variety of treatment specifications within the same stimulation protocols, and in many cases, conflicting results are reported. Thus, this systematic review aims to evaluate and critically discuss the existing evidence on these multi-mechanical wave-based stimuli and their impact on different aspects of AD pathology, but also on age-related impairments that often occur concomitantly with the development of AD and related dementia (e.g., impairments in neuromuscular function and mobility).

## Methods

This systematic review was conducted according to the Preferred Reporting Items for Systematic Reviews and Meta-Analyses (PRISMA) guidelines.

### Search strategy

The electronic search was performed in Scopus, PubMed, and Web of Science databases by the date of 26 December 2020. The following search terms were employed: ("Alzheimer's disease" OR "cognitive decline" OR “mild cognitive impairment”) AND (“music therapy” OR “acoustic wave” OR “acoustic stimulation” OR “auditory stimulation” OR “sound stimulation” OR “somatosensory stimulation” OR “whole-body vibration” OR “vibration therapy” OR “sound wave” OR “mechanical wave” OR “rhythmic sensory stimulation” OR “vibroacoustic therapy” OR “vibrotactile stimulation” OR “physioacoustic therapy” OR “binaural beats” OR “tone stimulation” OR “scanning ultrasound” OR “focused ultrasound” OR "sonication" OR “ultrasonic therapy”).

### Eligibility criteria

The established requirements for eligibility are depicted in Table 1. Titles and abstracts were screened and filtered according to the criteria.

**Table 1:** Eligibility criteria for admission in this review

Inclusion criteria	Exclusion criteria
Preclinical and clinical studies reporting noninvasive application of mechanical vibration-based approaches to impact AD pathology and/or related symptoms Preclinical and clinical studies reporting the application of mechanical vibration-based approaches to impact physical and/or cognitive deficits in aged animal models or non-demented older adults (aged over 60 years) Studies written in English	Reviews, conference papers, proceedings papers, editorials, and surveys Studies reporting the use of mechanical waves to facilitate/interfere with drug/gene delivery into the brain Studies in which mechanical stimulation is combined with other (unusual) interventions/activities (e.g., physical exercise) Studies in which nonperiodic mechanical waves are used to stimulate the brain Studies that evaluate the effect of mechanical stimulation in individuals suffering from cognitive decline derived from other diseases/conditions (e.g., stroke, ischemia, Parkinson’s disease)

### Quality assessment

In line with previous systematic reviews in AD [32, 33], the quality assessment of the reviewed clinical studies was performed using the Effective Public Health Practice Project (EPHPP) Quality Assessment Tool, and classified according to their global rating as having low (no “Weak” ratings), moderate (one “Weak” rating) or high (two or more “Weak” ratings) risk of bias [34]. For animal studies, the Stroke Therapy Academic Industry Roundtable (STAIR) preclinical recommendations were used [35]. Each study was assessed independently by two authors (FM and OC) and, when different results were achieved, the authors reevaluated the original paper until reaching a consensus. Complete quality assessment data, including the defined criteria, the results for each study, and their categorization, are provided as Additional file 1.

## Results

### Article selection and quality assessment

**Fig. 1:**
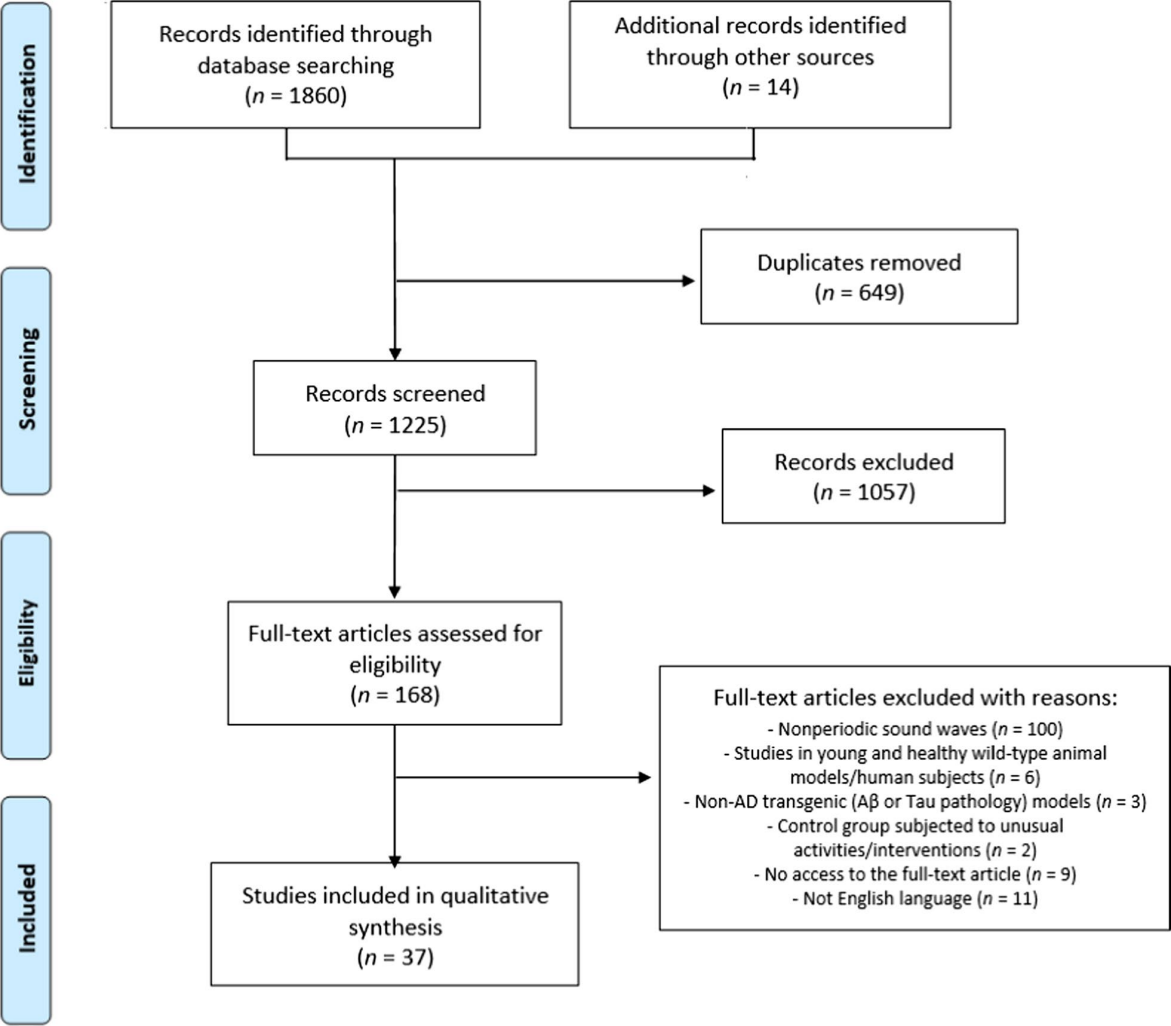
Flowchart of the article selection process. Adapted from [101]

The electronic literature search resulted in 1860 publications, and other 14 papers found in the literature review were added to the study list; then 649 duplicates were removed. By screening the titles and abstracts of the remaining 1225 titles, 168 full-text articles were assessed for eligibility, and 37 of them met the defined criteria and were included in the current systematic review. The article selection process is schematized in Fig. 1.

Concerning the methodological quality assessment, the EPHPP Quality Assessment Tool classified 17 clinical studies (*n*_WBV_ = 10, *n*_TUSS_ = 3, *n*_AS_ = 4) as having low risk of bias, three WBV and one TUSS studies were classified as having a moderate risk of bias, and only two studies presented a high risk of bias (two WBV papers). For the animal studies, the STAIR preclinical recommendations showed that the overall quality was not satisfactory; none of the included studies performed a sample size calculation, only one correctly described the method of allocation concealment [36], only two studies reported the reasons for animal exclusion from analysis [37, 38], and the majority of them did not mention the existence of any exclusion at all. Although many studies state that the animals were randomly allocated to the experimental groups, only one study provided details about the method of randomization [37]. The overall quality assessment data are presented in Additional file 1: Tables S1 and S2.

### Profile of the included articles

This systematic review focuses on both preclinical and clinical studies that used three types of mechanical vibration-based stimulation: WBV (*n* = 14, Table 2), TUSS (*n* = 17, Table 3), and AS (*n* = 6, Table 4).

**Table 2:** Summary of the selected whole-body vibration studies

Publication	Stimulation frequency	Amplitude	Duration and periodicity	Subjects under study	Primary outcomes of whole-body vibration (WBV) training
Kawanabe et al*.* [75]	12–20 Hz (rotational vibration)	–	4 min/day, once a week over 2 months	Healthy non-demented older adults (age: 71.8 ± 0.9)	***Behavioral readouts*** Improved walking ability overtime after WBV + routine exercise (10-m walking time and step length) Improved balance overtime after WBV + routine exercise (maximum standing time on one leg) No significant changes after routine exercise alone ***Safety and feasibility*** Well-tolerated by the elderly No serious adverse effects
Cheung et al*.* [74]	20 Hz (rotational vibration)	0.5-3 mm	3 min/day, 3 days/week over 3 months	Healthy non-demented older adults (age: 72.4 ± 4.9)	***Behavioral readouts*** Enhancement of some parameters of balance - e.g. movement velocity, maximum excursion and directional control (limits of stability assessment), and tendency for improvement in others - reaction time, endpoint excursion, and maximal distance reached while maintaining a fixed base of support (limits of stability assessment and functional reach test) ***Safety and feasibility*** Compliance of 93% Low level of dropouts (10%)
Furness and Maschette [73]	15 & 25 Hz (increasing over time) (vertical vibration)	0.05 mm	5 min (5 bouts of 1 min), over 6 weeks: once a week twice a week thrice a week (1–2 min interval between bouts)	Healthy non-demented older adults (age: 72 ± 8)	***Behavioral readouts*** Enhanced functional mobility after 2 and 3 WBV sessions/week (5-chair stands test and TUG tests) Improvement of balance and gait after 2 and 3 WBV sessions/week (Tinetti test) Increase in all health-related quality of life component scores after 2 and 3 WBV sessions/week (SF-36 score) ***Safety and feasibility*** Compliance of 100% Well-tolerated by the elderly No serious adverse effects
Cardinale et al*.* [100]	30 Hz (vertical vibration)	4 mm (peak-to-peak)	5 series of 1 min (1 min of rest between series)	Healthy non-demented older adults (age: 66–85)	***Cellular and molecular readouts*** Increased IGF-1 levels at the end of the treatment and at the 2-h follow-up (ELISA) Increased cortisol levels at the end of the treatment, with an abrupt decline at the 2-h follow-up (ELISA) No significant difference in growth hormone and testosterone levels compared to controls (ELISA) No changes in pulse rate and normal and spun haemoglobin (cyanmethaemoglobin method) ***Safety and feasibility*** Acceptability of 9 out of 10 (Likert scale)
Machado et al*.* [77]	20–40 Hz (increasing over time) (vertical vibration)	2-4 mm	7–22 min/session, 3–5 sessions/week over 10 weeks (increasing training volume over time)	Healthy non-demented older adults (age: 79.3 ± 7.3)	***Behavioral readouts*** Improvement of functional mobility (TUG test), associated with increased muscle strength, size, and activity in the lower limbs (maximal voluntary isometric contraction test, muscle cross-sectional area assessment, and surface electromyographic activity) compared to the control group and baseline condition ***Safety and feasibility*** Attendance of 95% No serious adverse effects
Marín et al*.* [79]	35 & 40 Hz (increasing over time) (vertical vibration)	1.05–2.11 mm (peak-to-peak)	- 2 sessions/week over 8 weeks - 4 sessions/week over 8 weeks	Healthy non-demented older adults (age: 84.3 ± 7.4)	***Behavioral readouts*** No alterations in balance (Romberg test) and health-related quality of life (SF-36 score) Increased neuromuscular function at the end of 2-sessions/week and 4-sessions/week regimens, and at the 3-weeks follow-up after the 4-sessions/week regimen (30-s chair stand test) ***Safety and feasibility*** No serious adverse effects
Beaudart et al*.* [102]	35 Hz (vertical vibration)	2 mm	5 series of 15 s (30 s of rest between series)	Healthy non-demented older adults (age: 82.2 ± 9.02)	***Behavioral readouts*** Tendency for improvement in balance and gait over time (Tinetti test) Tendency for improvement in functional mobility over time when data were adjusted according to subjects’ features (TUG test) No effect on the number of falls compared to the control group ***Safety and feasibility*** Attendance rate of 96.7%, with 91.9% compliance
Zhang et al. [76]	6–26 Hz (rotational vibration)	1-3 mm	4–5 min (4–5 bouts of 60 s), 3–5 times/ week over 8 weeks	Frail older adults without severe cognitive impairment (age: 85.3 ± 3.6)	***Behavioral readouts*** Enhanced balance, functional mobility, and physical functioning at week 4 (TUG test, knee extensor strength, surface area elipse, and SF-12 score) and at week 8 (TUG test, knee extensor strength, surface area elipse, and SF-12 score) ***Safety and feasibility*** High compliance Low level of dropouts (14%) No serious adverse effects
Sievänen et al. [81]	12 Hz, 18 Hz, and 26 Hz (rotational vibration)	2-8 mm (peak-to-peak)	1–5 min/session twice a week over 10 weeks (increasing training volume over weeks)	Frail older adults without severe cognitive nor functional impairment (age: 84.0 ± 7.4)	***Behavioral readouts*** No significant difference in physical performance, although some participants showed functional improvement (short physical performance battery scores, walking speed metrics, TUG and grip strength tests) ***Safety and feasibility*** Compliance and attendance of 74% (treated group) 12-Hz sessions were well tolerated by the elderly, but not the 18-/26-Hz ones No serious adverse effects
Álvarez-Barbosa et al*.* [78]	30–35 Hz (increasing over time) (vertical vibration)	4 mm (peak-to-peak)	12–17 min/session, 3 sessions/week over 8 weeks	Healthy non-demented older adults (age: 80–95)	***Behavioral readouts*** Enhanced functional mobility at the 4-, 6- and 8-weeks follow-up within the treatment (TUG test) Improved lower limb performance at the 2-, 4-, 6- and 8-weeks follow-up (30-s chair-sit-to-stand test) No changes in postural ability (Kistler force platform) Enhanced daily functioning and health-related quality of life (Barthel Index of activities of daily living and EuroQol-5D) ***Safety and feasibility*** Compliance of 73% (treated group) Well-tolerated by the elderly No serious adverse effects
Lam et al*.* [21]	30 Hz (vertical vibration)	2 mm (peak-to-peak)	- 4 min (8 bouts of 30 s), 2 days/week over 3 weeks (weeks 1–3) - 6 min (8 bouts of 45 s), 2 days/week over 5 weeks (weeks 4–9) - (1–2 min interval between bouts)	Demented patients *(mild or moderate dementia)* (age: 79.8 ± 6.1)	***Behavioral readouts*** Improved functional mobility at 3-month follow-up, but not at the end of the treatment (TUG test) Enhanced balance at the end of the treatment and at the 3-month follow-up (Berg Balance Scale, and Tinetti balance assessment) Quality of life improvement at the end, but not at the 3-month follow-up (Quality of Life in Alzheimer’s disease score) Lower functional status at baseline was associated with greater improvement in physical function (TUG , 5-time-sit-to-stand, Berg Balance Scale, Tinetti balance tests) ***Safety and feasibility*** 86% compliance (intention-to-treat analysis) Two out of 27 participants reported mild knee pain and no other side effects were reported
Kim and Lee [67]	20–40 Hz (increasing over time) (vertical vibration)	-	5 times/week over 8 weeks	Senile-demented women *(mild dementia)* (age: 79.2 ± 4.0)	***Brain function and behavioral readouts*** Increased brain network activation over time (electroencephalographic signal) Enhanced cognitive function (Mini mental state examination)
Zhu et al*.* [72]	12–16 Hz (rotational vibration)	3-5 mm (peak-to-peak)	20 min/day for 5 days over 8 weeks	Sarcopenic non-demented older adults (age: 89.5 ± 4.4)	***Behavioral readouts*** No differences in muscle mass (dual-energy X-ray absorptiometry) Increased handgrip and lower-limbs strength over time (handheld JAMAR and FET3 dynamometers) Improved overall physical performance over time (6-m gait speed test, TUG, 5-times-sit-to-stand, and balance tests)
Heesterbeek et al*.* [84]	30 Hz	1-2 mm	4 min, 4 days/week over 6 consecutive weeks	Demented patients *(any form of dementia)* (age: 85.3 ± 6.8)	***Safety and feasibility*** 83% attendance, 100% compliance, and an experience score of 6.9/10 (intention-to-treat analysis) 90% attendance, 100% compliance, and an experience score of 7.4/10 (per protocol analysis) No serious adverse effects Low level of dropouts (17%) Treatment classified as pleasant by the participants

WBV: whole-body vibration; TUG: time up and go; SF-n: short form health survey (n represents the number of questions in the questionnaire); IGF-1: insulin-like growth factor 1; ELISA: enzyme-linked immunosorbent assay

**Table 3:** Summary of the selected transcranial ultrasound stimulation studies

Publication	Center frequency	Duration and periodicity	Model/subjects under study	Delivery mode	Primary outcomes of transcranial ultrasound stimulation (TUSS)
Jordão et al*.* [59]	0.5 MHz (pulsing at 1 Hz)	Single session of 10-ms bursts for 2 min/spot (4 spots)	TgCRND8 mice *(4 months old)*	Transcranial (unilateral target)	***Cellular and molecular readouts*** Reduced Aβ plaque size in the treated cortex at the 4-days follow-up (IHC) Tendency to reduction of Aβ plaques number in the treated cortex at the 4-day follow-up (IHC) Enhanced interaction between endogenous antibodies and Aβ plaques in the treated cortex at the 4-day follow-up (IHC and confocal microscopy) Improved bioavailability of endogenous antibodies in the treated hemisphere at the 4-day follow-up (IHC and immunoblotting) Increased glial cell activity in the treated hemisphere at the 4-h, 4-day and 15-day follow-ups (IHC and WB) Increased Aβ internalization by glial cells at the 4-day follow-up in both treated and non-treated cortices, more marked in the treated hemisphere (IHC and confocal microscopy) ***Safety and feasibility*** Safe US-mediated BBB opening No adverse effects
Burgess et al*.* 2014 [38]	1.68 MHz (pulsing at 1 Hz)	10-ms bursts for 2 min (2 spots/ hemisphere), once a week over 3 weeks	TgCRND8 mice *(7 months old)*	Transcranial (bilateral hippocampi)	***Cellular and molecular readouts*** Reduced Aβ plaque size and number in the HPC (IHC) Increased number of immature hippocampal neurons, increased dendrite length and branching in pre-existing neurons of HPC (IHC) Behavioral readouts Improved spontaneous alternation (short-term memory) and exploratory skills (Y-maze test) ***Safety and feasibility*** Safe US-mediated BBB opening Well-tolerated by mice
Leinenga and Götz 2015 [36]	1 MHz (pulsing at 10 Hz)	6 s per spot (spots 1.5-mm apart through the entire brain) Over 4 weeks (sessions with 2-weeks interval), followed by a final US session (5 sonications in total) Weekly sessions over 7 weeks	Aged APP23 mice (*in vivo*) Microglial cells exposed to Aβ_42_ (*in vitro*)	Transcranial (whole brain)	***Cellular and molecular readouts*** Reduced Aβ plaque area and number in mice after the 4-weeks + 1 session regimen (IHC and HC) Reduced soluble and oligomeric Aβ species in the right hemisphere of mice after the 4-weeks + 1 session regimen (WB and ELISA) Reduced Aβ1-42 levels (ELISA andWB) in the brains of mice after the 7-week regimen Activation of microglia in the brain after the 7-week regimen (IHC) Increased internalization of Aβ by microglia in mice after the 4-weeks + 1 session regimen (spinning disk confocal microscopy and high-resolution 3D reconstruction) Increased Aβ_42_ uptake by microglial cells in the presence of albumin after the 7-weeks regimen (IHC and confocal microscopy) No significant differences in Tau hyperphosphorylation load after the 7-week regimen (WB) No differences in astrocyte immunoreactivity after the 7-week regimen (IHC) ***Behavioral readouts*** Improved spontaneous alternation (short-term memory) after the 4-week regimen (Y-maze) Enhanced spatial learning, long- and short-term memory after the 7-week regimen (active avoidance test) Improved recognition memory after the 7-week regimen (novel object recognition) ***Safety and feasibility*** No adverse effects
O’Reilly et al*.* 2017 [62]	0.28 MHz (pulsing at 1 Hz)	- Single session of 60 10-ms bursts/spot for 3 min; - 120 10-ms bursts/spot for 2 min (5-min interval), weekly over 4 weeks	Aged beagle dogs (Aβ-positive) *(9–11 years old)*	Transcranial (whole-left hemisphere)	***Cellular and molecular readouts*** No significant differences in Aβ load between hemispheres after both regimens (IHC) Tendency to reduced Aβ load in the left cortex compared to the ipsilateral area after both regimens (IHC) No significant differences in microglia activity between hemispheres after both regimens (IHC) ***Behavioral readouts*** No changes in gait, postural reaction or cranial nerve test after both regimens ***Safety and feasibility*** Intact BBB and no observed brain damage in all animals one week after both treatments (MRI) One dog suffered from thermal damage due to too high-pressure exposures, with no other adverse events
Leinenga and Götz [60]	1 MHz (pulsing at 10 Hz)	4 sessions of 2.4 min each (over 24 spots)	Aged APP23 mice *(21–22 months old)*	Transcranial (whole brain)	***Cellular and molecular readout*** No significant differences in Aβ plaque area and number in the forebrain after sonication (IHC) Reduced Aβ plaque area in the forebrain (IHC) Increased Aβ-associated microglia, mainly in larger plaques (IHC) **Safety and feasibility** Safe US-mediated BBB opening
Eguchi et al*.* 2018 [57]	1.875 MHz (pulsing at 6 kHz)	20 min/spot (3 spots), 3 sessions on days 1, 3, 5, 28, 30, 32, 56, 58, 60, 84 and 86 (over 12 weeks)	5xFAD mice *(14–16 weeks old)*	Transcranial (whole brain)	***Cellular and molecular readout*** Regulation of immune-related genes (RNA-sequencing) Reduced microgliosis in the cortex, despite increased microglia phagocytosis towards Aβ deposits (IHC) Upregulation of endothelial nitric oxide synthase, neurotrophins, and heat-shock protein 90 (WB) Downregulation of amyloid precursor protein and BACE-1 (WB) Reduced Aβ load and plaque burden throughout the brain (IHC and ELISA) Enhanced cerebral blood flow for up to 84 days after sonication (laser speckle blood flow imager) ***Behavioral readouts*** Improved memory performance in the spontaneous alternation task (Y-maze test) ***Safety and feasibility*** No adverse effects
Poon et al*.* 2018 [37]	- 1.1 MHz (pulsing at 1 Hz) - 1.68 MHz (pulsing at 1 Hz)	10-ms bursts for 2 min: - in a single session - once every 2 weeks, over 10 weeks	TgCRND8 mice *(6 months old)*	Transcranial (bilateral hippocampi)	***Cellular and molecular readouts*** Reduced Aβ plaque volume after one session of US until 14-day follow-up (two-photon fluorescence microscopy) Reduced Aβ plaque maximum cross-sectional area after one session of US until 7-day follow-up (two-photon fluorescence microscopy) Decreased Aβ plaque number and surface area in the HPC after 10-week treatment (IHC) ***Safety and feasibility*** Safe US-mediated BBB opening after both regimens (two-photon fluorescence microscopy and MRI)
Pandit, Leinenga & Götz 2019 [66]	1 MHz (pulsing at 10 Hz)	2.4 min (over 24 spots), once a week over 15 weeks	K3 mice *(6 weeks old)*	Transcranial (whole brain)	***Cellular and molecular readouts*** Tendency for reduced p-Tau levels in hippocampal slices Reduced p-Tau levels in the HPC, but not in the cortex (IHC) Reduced neurofibrillary tangles in the HPC and cortex (HC and IHC) Activation of microglia Increased autophagy in the neurons (IHC, WB, and proximity ligation assays) Behavioral readouts Improved motor ability and coordination, but no increased grip strength (repeated Rotarod paradigm) Enhanced spatial working memory (Y-maze test) Tendency for improved short-term memory (novel object recognition test) ***Safety and feasibility*** Safe US-mediated BBB opening, as no differences in opening volume occurred (MRI) Well-tolerated by mice
Karakatsani et al*.* 2019 [45]	1.5 MHz (pulsing at 10 Hz)	single session of 60 s	rTg4510 mice (Tau pathology model) *(3.5–4.5 months old)*	Transcranial (unilateral target)	***Cellular and molecular readouts*** Reduced p-Tau in the HPC of both hemispheres mainly in the treated one (IHC) Increased immune cells activation in both hemispheres compared to untreated brains (IHC) Fragments of p-Tau were found within microglia in both hemispheres (IHC) Increased immune cell activation was correlated with p-Tau reduction ***Safety and feasibility*** Safe US-mediated BBB opening, as no differences in opening volume occurred (MRI)
Bobola et al*.* [61]	2 MHz (pulsing at 40 Hz)	- Single session of 1 h (acute stimulation) - 1 h/day over 5 days (chronic stimulation)	5xFAD mice *(6 months old)*	Transcranial (left hippocampus for acute stimulation and bilateral hippocampi for chronic stimulation)	***Cellular and molecular readouts*** No significant differences in endothelial nitric oxide synthase production after chronic stimulation (histological analysis) Activation of microglia that co-localized with Aβ plaques after acute stimulation in the treated hemisphere (histological analysis) No significant changes in Aβ plaque load after acute stimulation in both hemispheres (IHC) Activation of microglia that co-localized with Aβ plaques after chronic stimulation in the treated brains Reduction in Aβ plaque burden after chronic stimulation (histological analysis) ***Brain function readouts*** Strong and temporally non-uniform signal entrainment at 40 Hz during acute stimulation (continuous wavelet transforms analysis of EEG signal) ***Safety and feasibility*** No side effects after treatment
Shen et al*.* [58]	0.996 MHz (pulsing at 1 Hz)	60-s sessions, twice a week over 6 weeks	3xTg-AD mice *(8 months old)*	Transcranial (unilateral target)	***Cellular and molecular readouts*** Reduced Aβ pathology in the cortex, amygdala, CA1 and CA3 hippocampal regions of the treated hemisphere (IHC) Decreased p-Tau levels in the cortex, HPC, and amygdala (IHC) Improvement of axonal neurofilament degeneration in the HPC of treated mice (confocal microscopy) Activation of microglial phagocytosis and extensive internalization of Aβ aggregates (confocal microscopy) Reduced microglia branching in the stratum radiatum layer in the treated hemisphere near Aβ deposits (confocal microscopy) Modulation of the expression of synaptic, microtubule, mitochondrial, glycolytic, and ubiquitin proteins (2D fluorescence difference gel electrophoresis combined with mass spectroscopy) ***Behavioral readouts*** Improved spatial learning as well as short- and long-term memory skills (Y maze, MWM, and step-down passive avoidance test) ***Safety and feasibility*** Well-tolerated by mice No neuron or tissue damage in the brain Safe and reversible US-mediated BBB opening (fluorescence imaging)
Lee et al*.* [56]	715 kHz (pulsing at 1 Hz)	Single session with 20-ms bursts delivered over 60 s	5xFAD mice *(4 months old)*	Transcranial (unilateral target)	***Cellular and molecular readouts*** Reduced total area of Aβ deposits, but not Aβ plaques, in both treated and non-treated hemispheres (IHC) Reduced gliosis in the HPC and entorhinal cortex in both treated and non-treated hemispheres (IHC) Increased Aβ species around meningeal vessels (IHC) Increased microglia around Aβ plaques (IHC) Increased drainage of soluble Aβ to the cerebrospinal fluid space in TUSS-treated mice in which ligation of lymphatics to the deep cervical lymph nodes was performed (IHC) Prevention of neuronal loss and glial cell reactivity/activation in the entorhinal cortex, but not in other brain areas (H&E and TUNEL staining) ***Behavioral readouts*** Improved working memory, with no alterations in mobility (Y maze) ***Safety and feasibility*** Well-tolerated by mice No neuron or tissue damage in the brain Safe and reversible US-mediated BBB opening (fluorescence imaging)
Lipsman et al*.* [63]	220 kHz (continuous mode)	7.5 min s/session, along 3.6 sessions (stage 1), followed by 7.5 sessions (stage 2) over 3 months, with 1-month interval between stages	AD patients *(mild to moderate AD)* (age: 66.2 ± 6.6)	Transcranial (whole brain)	***Cellular and molecular readouts*** No significant differences in Aβ levels ([^18^F]-florbetaben PET scans) ***Behavioral readouts*** No changes in cognition and daily functioning at the 3-month follow-up (MMSE, ADAS-cog, ADCS-ADL, GDS, and NPI-Q) One of the patients showed a transient cognitive improvement in the 1-month follow-up after stage 2 (NPI-Q) ***Safety and feasibility:*** Safe, temporary and repeatable USS-mediated opening, even in Aβ-rich brain regions (MRI) No serious adverse events
Meng et al*.* [68]	220 kHz (continuous mode)	Two sessions, one month apart, doubling the target volume in the second intervention	AD patients *(mild to moderate AD)* (age: 66.8 ± 6.1)	Transcranial (right frontal lobe)	***Brain function readouts*** Decreased functional connectivity in the right frontoparietal networks during BBB opening, which was reversed at 1-day and 1-week follow-up (resting-state BOLD fMRI signal) Tendency for decreased functional connectivity of the right frontoparietal network at the 3-month follow-up compared to baseline, although the US inhibited the marked decrease that occurred in the control group (resting-state BOLD fMRI signal) Tendency for increased functional connectivity of the default mode network at the 3-month follow-up in US-treated patients compared to controls (resting-state BOLD fMRI signal) ***Safety and feasibility*** Safe, effective, and reversible US-mediated BBB opening (MRI)
Beisteiner et al*.* [69]	- (pulsing at 5 Hz)	2 spots per session, 3 sessions/week over 2–4 weeks	AD patients	Transcranial (bilateral target)	***Behavioral and brain function readouts*** Improved cognitive status after treatment and at the 3-month follow-up improved memory and verbal processing and decline in visuospatial processing (CERAD scores and SEG scale) Upregulation of memory network after treatment (fMRI) Increased functional connectivity in HPC, parahippocampal cortex, parietal cortex and precuneus (resting-state fMRI) and bilateral HPC (task-based fMRI) Improved brain functional connectivity that was correlated with cognitive improvements (CERAD scores) ***Safety and feasibility*** No side effects after treatment and at the 3-month follow-up (clinical assessments, patients reports, and MRI)
Rezai et al*.* [82]	220 kHz (continuous mode)	2–5 sonications over 3 sessions separated by 2 weeks	AD patients *(early AD)* (age: 55–75)	Transcranial (bilateral hippocampi/ entorhinal cortex)	***Behavioral readouts*** No significant changes in cognitive function at the 30-day follow-up (formal cognitive assessments) Safety and feasibility Well-tolerated by the patients No side effects after treatment Safe, feasible, reversible, reproducible, focused, and noninvasive US-mediated BBB opening in the HPC and entorhinal cortex (MRI)
D’Haese et al*.* [65]	220 kHz (continuous mode)	3 sessions separated by 2 weeks	AD patients *(early AD)* (age: 55–73)	Transcranial (unilateral hippocampi/ entorhinal cortex)	***Cellular and molecular readouts*** Reduced Aβ plaques in the stratum radiatum layer in the HPC and entorhinal cortex of the treated hemisphere ([^18^F]-florbetaben PET scans) ***Behavioral readouts*** No significant changes in cognitive function at the 1-week follow-up (formal cognitive assessments) ***Safety and feasibility*** Safe, feasible and reversible US-mediated BBB opening in the HPC and entorhinal cortex (MRI)

Tg: transgenic; Aβ: β-amyloid; IHC: immunohistochemistry; WB: Western blot; US: ultrasound; ; BBB: blood–brain barrier; AlCl_3_: aluminum chloride; MWM: Morris water maze; HPC: hippocampus; ELISA: enzyme-linked immunosorbent assay; p-Tau: phosphorylated Tau; MRI: magnetic resonance imaging; EGR1: early growth response protein 1; *CA1, CA3: Cornu Ammonis* hippocampal areas; *DG:* dentate gyrus; AChE: acetylcholinesterase; BrdU: bromodeoxyuridine; BDNF: brain-derived neurotrophic factor; RT-PCR: real-time polymerase chain reaction; PET: positron emission tomography; MMSE: mini-mental state examination, ADAS-cog: Alzheimer’s disease assessment scale – cognitive; ADCS-ADL: Alzheimer’s disease cooperative study group—activities of daily living; GDS: geriatric depression scale; NPI-Q: neuropsychiatric inventory questionnaire; BOLD: blood-oxygenation-level-dependent; fMRI: functional MRI; CERAD: consortium to establish a registry for Alzheimer's disease; SEG: scale for subjective evaluation of memory performance (translated from German)

**Table 4:** Summary of the selected auditory stimulation studies

Publication	Intermittence frequency	Duration and periodicity	Model/ Subjects under study	Primary outcomes of auditory stimulation (AS)
Lee et al*.* [48]	40 Hz	2 h/day over 2 weeks	5xFAD mice *(5 months old)*	***Molecular and cellular readouts*** Reduced Aβ plaque number in the prelimbic and infralimbic cortices and HPC (IHC) Reduced soluble and insoluble Aβ_1-40_ and Aβ_1-42_ species in the prelimbic and infralimbic regions (ELISA) Increased microglia number in the HPC and prelimbic and infralimbic cortices (IHC) ***Brain function readouts*** Exaggerated increase of evoked gamma band oscillations in the day after stimulation, decreased over the treatment (wavelet analysis of EEG) Increased resting-spontaneous gamma power on days 7 and 14 (EEG) Evoked gamma oscillation in the brain facilitates microglial aggregation in 5xFAD mice and their wild-type littermates (EEG and IHC) Enhanced brain connectivity, with frontal gamma rhythms coupling with parietal delta at baseline; and to parietal theta brainwaves after stimulation (EEG)
Martorell et al*.* [30]	- 8, 40, 80 Hz and random tone stimulation *(AS)* - 40 Hz and random tone stimulation *(AS* + *VS)*	20 min/session (1–10 min intervals) 1 h/day (5 sessions) over 7 days	5xFAD *(6 months old)* C57BL/6 J *(3–8 months old)* APP/PS1 *(6 months old)* Tau P301S mice *(6 months old)*	***Molecular and cellular readouts*** Reduced soluble Aβ levels and Aβ plaque area and number in the AC and HPC of 5xFAD and APP/PS1 mice after 40-Hz AS (ELISA and IHC) Increased microglia response in the AC and HPC of 5XFAD and APP/PS1 mice after 40-Hz AS, reversed at the 1-week follow-up, except for microglia number in the AC of 5xFAD mice (IHC) Increased astrocyte reactivity and vascular dilation in the AC and HPC of 5XFAD mice after 40-Hz AS (IHC) Reduced Tau hyperphosphorylation and seeding in the AC and HPC of Tau P301S mice after 40-Hz AS (IHC, WB, and FRET) Increased microglial activity displaying an encapsulating effect around Aβ aggregates in the AC, HPC, and mPFC after 40-Hz AS + VS (IHC and 3D reconstruction using IMARIS) Reduced Aβ plaque area and number in the AC, VC, HPC, and mPFC of 5XFAD mice after 40-Hz AS + VS (IHC and SHIELD processing) Decreased soluble and insoluble Aβ_1-42_ levels in the AC, VC, HPC, and mPFC of 5XFAD mice after 40-Hz AS + VS (ELISA and IHC) ***Behavioral and brain function readouts:*** Improved spatial and recognition memory after 40-Hz AS in 5xFAD mice (MWM, novel object recognition, and novel object location tasks) Gamma brainwave entrainment in the AC and HPC after 40-Hz AS (electrophysiology) Gamma brainwave entrainment in the AC, HPC, and mPFC after 40-Hz AS + VS (electrophysiology) Greater entrainment and therapeutic effects after combined AS + VS compared to AS alone
Clements-Cortes et al*.* [83]	40 Hz (VS + VAS)	30 min/session, 2 sessions/week over 6 weeks (6 VS and 6 VAS sessions)	AD patients *(mild to severe AD)* (age: 59–93)	***Behavioral readouts*** Increased cognitive function after 40-Hz VAS, but not after VS (Saint Louis University mental status) No significant changes in mood and anxiety (observed emotion rating scale) Increased awareness of surroundings, increased interaction, stimulation of discussion/story telling, and increased alertness after 40-Hz VAS (researcher observation)
Calomeni et al*.* [49]	8, 10, 12, 14, and 15 Hz ***(AS* + *VS)***	3 min for each frequency/day, 10 sessions on alternate days over 20 days	AD patients and healthy non-demented older adults (age: 76 ± 8)	***Behavioral and brain function readouts*** Gains in memory function associated with brain wave modulation (EEG) Enhanced alpha brainwave activity in AD patients, but not in non-demented elderly (EEG) Tendency for improved working memory function in AD patients (Digit Span Test)
Papalambros et al*.* [70]	20 Hz	Five 50-ms pulses (1.2-s interval) over one night during sleep	Healthy non-demented older adults (age: 60–84)	***Behavioral and brain function readouts*** Increased slow-wave activity and tendency for improved theta and fast spindle power during stimulation (EEG) Enhanced amplitude during stimulation for all potentials and larger slow-waves (EEG) Increased spindle density and amplitude during stimulation (EEG) Increased slow-wave activity during stimulation was associated with improved memory function (cued-recall tests) Mean phase stimulation was positively correlated with recall enhancement No significant differences in self-reported sleep quality, mood, and alertness between stimulated and sham-treated subjects (sleep-quality, mood and alertness questionnaires)
Papalambros et al*.* [31]	20 Hz	Five 50-ms pulses (1.2-s interval) over one night during sleep	Amnestic mild cognitive impaired patients (age: 72 ± 8.7)	***Behavioral and brain function readouts*** Increased slow oscillations, slow-wave, and sigma activities during stimulation (EEG) No significant differences in theta and beta activities during stimulation (EEG) Tendency for improved overnight word recall after stimulation (cued-recall tests) Alterations in slow-wave, slow oscillations, and sigma activities during stimulation were positively correlated with overnight word recall Increased slow-wave activity during stimulation was associated with improved cognition (verbal paired-associates task and NIH Toolbox Cognition Battery) Improved memory in 5 out of 9 patients (cued-recall tests) No significant differences in self-reported sleep quality (sleep quality and alertness questionnaires) and sleep-staging features between stimulated and sham (polysomnography)

Aβ: β-amyloid; IHC: immunohistochemistry; ELISA: enzyme-linked immunosorbent assay;EEG: electroencephalography*;* VS: visual stimulation; AC: auditory cortex; HPC: hippocampus; FRET: Förster resonance energy transfer*;* mPFC: medial prefrontal cortex; VC: visual cortex; MWM: Morris water maze; VAS: vibroacoustic stimulation; AD: Alzheimer’s disease

At the clinical level, the effect of WBV training was assessed in cohorts of non-demented older adults (*n* = 11) or individuals diagnosed with mild or moderate dementia (*n* = 3) using different cognitive status tests such as Cantonese mini-mental state examination and Mini-Mental State Examination.

The TUSS and AS studies included in this systematic review describe both preclinical and clinical evidence. Regarding the 17 TUSS studies, five of them were dedicated to the investigation of the effectiveness of combined ultrasound treatment and microbubble injection in AD patients; the remaining TUSS studies were conducted in AD transgenic mouse models (*n* = 11) and aged amyloid-β (Aβ)-positive dogs (*n* = 1).

For AS papers, two animal studies were performed in AD transgenic mice using periodic stimuli while one of them combined AS with visual stimulation. The remaining four AS studies were performed in humans using heterogeneous protocols; one study provided vibroacoustic stimulation (i.e., therapeutic strategy using vibratory sound stimuli) to AD patients; another study tested the effect of combined auditory and visual stimuli in AD patients, and the other two studies described the implementation of an AS protocol during sleep in amnestic mild cognitive impairment (MCI) patients (i.e. a prodromal stage of AD) and healthy non-demented older subjects.

The data collected from the included studies are organized in Tables 2, 3 and 4, summarizing the implemented protocols and the most important findings from the WBV, TUSS, and AS studies, respectively. The studies are compiled in chronological order, starting with animal studies, followed by clinical trials. The readouts used in the original papers are categorized and compared in Table 5.

**Table 5:** List of the aspects of AD pathology in which mechanical-based modality produced positive effects for each of the reviewed studies

	Aβ and/or Tau pathologies	Immunological response	Neurotoxicity and neuroplasticity deficits	Brainwave entrainment and brain functional connectivity	Behavioral performance	
					Cognitive and mood status	Motor ability
***Whole-body vibration***						
Kawanable et al*.* [75]						X
Cheung et al*.* [74]						X
Furness and Maschette [73]						X
Cardinale et al*.* [100]						X
Machado et al*.* [77]						X
Marín et al*.* [79]						X
Beaudart et al*.* [102]						
Zhang et al*.* [76]						X
Sievänen et al. [81]						
Álvarez-Barbosa et al*.* [78]						X
Lam et al*.* [21]						X
Kim and Lee [67]				X	X	
Zhu et al*.* [72]						X
Heesterbeek et al*.* [84]						
***Transcranial ultrasound stimulation***						
Jordão et al*.* [59]	X	X				
Burgess et al*.* [38]	X		X		X	
Leinenga and Götz [36]	X	X			X	
O’Reilly et al*.* [62]						
Leinenga and Götz [60]	X	X				
Eguchi et al*.* [57]	X	X	X		X	
Poon et al*.* [37]	X					
Pandit et al*.* [66]	X	X	X		X	
Karakatsani et al*.* [45]	X	X	X			
Bobola et al*.* [61]	X	X		X		
Shen et al*.* [58]	X	X	X		X	
Lee et al*.* [56]	X	X	X		X	
Lipsman et al*.* [63]					X	
Meng et al*.* [68]				X		
Beisteiner et al*.* [69]				X	X	
Rezai et al*.* [82]						
D’Haese et al*.* [65]	X					
***Auditory stimulation***						
Lee et al*.* [48]	X	X		X		
Martorell et al*.* [30]	X	X		X	X	
Clements-Cortes et al*.* [83]					X	
Calomeni et al*.* [49]				X	X	
Papalambros et al*.* [70]				X	X	
Papalambros et al*.* [31]				X	X	

### Basic principles of the three mechanical-based modalities and their relationship to brain pathology

WBV training consists of passive exercise training delivered by a vibratory platform in which  high-frequency (5–40 Hz) and small-amplitude mechanical stimuli are applied to the entire body [13, 14]. During WBV training, the vibration delivered by the oscillating platform stimulates the neuromuscular system by activating muscles through spinal reflexes and increasing synchronous motor unit recruitment [39]. These phenomena enhance neuromuscular excitability, inducing muscle contractions [39, 40], which has been shown to produce numerous beneficial effects in older adults on neuromuscular function [15–17], vascular diseases [18], and neurological conditions [19, 20]. However, despite the wide range of clinical application, limited doses of WBV are well tolerated by the elderly, as overexposure to WBV training can result in over-stress injuries [14]. On the other hand, insufficient stimulation may result in the lack of the desired improvement. For this reason, the dose–response relationship for WBV needs to be carefully defined for each application or individual (including an AD patient-tailored procedure), and no optimal protocols have yet been established.

TUSS is a modality used in combination with microbubble injection, which transiently opens the blood–brain barrier (BBB). After intravenous injection in the brain, the ultrasound (US)-sonicated microbubbles undergo expansion–contraction cycles that disrupt the tight junctions along the blood vessel walls, creating new entries through the BBB [41], usually reversed within 24 h. Importantly, the size and dose of microbubbles, as well as their manipulation and synchronization with sonication must be appropriate to allow the efficiency and success of the process. Also, ultrasonic waves are mainly characterized *via* the center frequency (associated with the energy carried by the waves), the emission mode (i.e., continuous or pulsed mode), and the power density or pressure that is applied to a certain body/surface (associated with the intensity of the stimulation). The TUSS microbubble-assisted approach has been widely used in the last decade to facilitate the delivery of therapeutic agents for the treatment of a wide range of neurological disorders, showing promising efficiency to facilitate their pharmacological effect [42–45]. However, recent studies have shown the beneficial action of TUSS (i.e. without association to any drug) to stimulate neurogenesis, neuronal excitability, and/or brain network activity in wild-type experimental animals [23–29] and improve AD brain pathology and related memory deficits in AD mouse models [23–29, 46, 47]. These achievements have sparked clinical interest in the pursuit of an effective noninvasive intervention based on the transcranial application of US to ameliorate AD pathology. Yet, the novelty of this technique entails many doubts concerning the optimal protocols and the long-term effects as discussed below (see Section Limitations and future directions).

More recently, AS has been receiving increased attention due to its effectiveness in reducing Aβ and Tau pathology and improving cognitive abilities in AD animal models [30, 48]. This is a sensory-based stimulation strategy delivered by trains of tones repeated at a specific frequency which has been associated with marked entrainment of brainwaves in the brains of AD transgenic mice [30, 48] and AD patients [31, 49]. In fact, Aβ and Tau pathologies are known to alter brain rhythmic oscillatory activities [50], mainly in high-frequency band waves, impacting the high-order cognitive domains [51, 52]. High-frequency brainwaves, e.g. gamma oscillations (30–100 Hz), are disrupted in both AD patients and AD transgenic mouse models [51, 53]. During the last years, sensory stimulation has been used to affect/stimulate gamma band oscillations in the brain. Importantly, this brainwave entrainment has been correlated with the attenuation of AD pathomechanisms in AD mouse models [51, 54], as well as with the improvement of cognitive function and sleep disturbances in younger adults [55].

### Impact on AD pathomechanisms and associated brain malfunction

This section presents and discusses findings from studies on the three modalities (Tables 2, 3, 4), classified into cellular, molecular, brain function/connectivity and behavioral outputs.

#### Aβ and Tau pathologies

In WBV studies, no biochemical or brain imaging analysis was performed to assess possible alterations of Aβ and/or Tau pathology in the brains of participants. On the other hand, the reviewed studies on TUSS and AS provide plenty of preclinical evidence on their effectiveness to reduce/block Aβ and Tau accumulation and aggregation.

##### TUSS

Ultrasound pulses delivered transcranially inhibit the formation of Aβ plaques by decreasing soluble Aβ species, as shown in the brains of three different AD transgenic mouse models with advanced Aβ pathology, i.e. APP23, 3xTg, and 5xFAD [36, 56–58]. In some cases, pulsed TUSS is able to markedly reduce Aβ plaque load (affecting number, size, area) in different AD transgenic mice after single [37, 59] or multiple US sessions [36, 37, 57, 58, 60, 61]. It should be noted that these studies have typically used similar stimulation frequencies (center frequency ~ 1 MHz, pulsing at 1–10 Hz), but very discrepant duration (few minutes to 1 h for each session, delivered either in a single application or over several weeks). Moreover, two studies in Aβ-based transgenic mice (i.e., TgCRND8) with abundant amyloid plaque load reported that part of the TUSS beneficial effect was lost when follow-up periods were considered; both Jordão et al. (2013) and Poon et al. (2018) reported that the reduction of Aβ plaque area was observed four to seven days after mouse brain stimulation, but this recovery was lost two weeks after a single session of 2 min [37] or 8 min [59]. Another study in aged Aβ-positive dogs showed a tendency for reduced Aβ load in the prefrontal cortex of the US-treated hemisphere, in both single- and multiple-session interventions [62]. This limited impact of TUSS treatment on Aβ load could be attributed to the insufficient stimulus duration or intensity (such as insufficient duration of each stimulation, e.g. 2 min stimulation once a week, 4 weeks period, or insufficient ultrasonic intensity, e.g. 0.28 MHz pulsing at 1 Hz, as the center frequency is relatively lower compared to all the other preclinical experiments in AD transgenic mice). Another parameter to be considered is the advanced state of Aβ pathology of the experimental animals (dogs in the above study), which may suggest that such applications should be more beneficial if they are offered in early stages of AD brain pathology [62].

At the clinical level, only two studies have investigated the effect of TUSS on Aβ in the brains of mild-to-moderate AD patients (aged 57–73 years), by using [^18^F]-florbetaben PET scan [63, 64]. However, conflicting outcomes have been reported. The first study by Lipsman and colleagues (2018) found no significant alterations in Aβ level after TUSS treatment (7.5 min once a week, 3 months period) delivered using a helmet-like device (whole-brain stimulation) in mild-to-moderate AD patients [63]. On the contrary, D’Haese et al*.* (2020) reported a reduction of Aβ plaques in the hippocampus and entorhinal cortex of the treated hemisphere after unilateral stimulation (3 sessions, 6 weeks period) in patients at early stage of AD and at a similar age [65].

Although less studied, the beneficial effect of TUSS on Tau hyperphosphorylation and aggregation has recently emerged. Experimental studies have described a TUSS-driven decrease of p-Tau levels in the hippocampus of three different mutant-Tau transgenic mouse models (i.e., K3, rTg4510, and 3xTg-AD mice) after single [45, 58] or multiple US sonications (2.4 min once a week, 15 weeks period) [66]. Reduction is also observed for neurofibrillary tangles, the final intraneuronal aggregates of Tau, in K3 mice [66]. Interestingly, Karakatsani et al*.* (2019) observed a reduction of p-Tau in both hemispheres of 4-month-old rTg4510 mouse brain after a single session of unilateral-target US sonication [45], showing that US is capable of producing a widespread and systemic response across the brain beyond the sonicated areas, even with a minimum dose of TUSS (1-min session).

##### AS

For AS, only preclinical studies have monitored its effect on Aβ load, using trains of tones repeating at 40 Hz. In the study conducted by Martorell et al. (2019), the trains of acoustic tones (1 h/day, 7 days period) evoked the clearance of Aβ soluble species and the decrease of Aβ plaques in the auditory cortex and hippocampus of 5xFAD and APP/PS1 mice; both mouse models exhibit marked Aβ plaque burden [30]. Similar results were found in another study [48] that used a longer stimulation protocol (2 h/day, 15 days period) and reported that 40-Hz AS stimulation decreased both soluble and insoluble Aβ species, accompanied by a reduction in Aβ plaque number throughout the brains of 5xFAD mice [48]. Interestingly, when AS was combined with another sensory modality (visual stimulation using light flickering at the same rate—40 Hz), a wider effect was produced across the brain, which exhibited reduced Aβ pathology not only in the auditory cortex and hippocampus but also in the medial prefrontal cortex of 5xFAD mice [30].

In addition, only one study has reported a beneficial impact of AS on Tau pathology. By applying 40-Hz AS, Martorell and his collaborators (2019) observed a reduction in Tau hyperphosphorylation and seeding in the auditory cortex and hippocampus of P301S-Tau transgenic mice (1 h/day, 7 days period) [30]. No clinical evidence exists on the effectiveness of mechanical vibrations using any of the reviewed stimulation modes in mitigating Tau phosphorylation and accumulation in the AD human brain.

#### Immunological response

Among the reviewed studies performed in experimental models, 11 out of 14 have reported a positive immunological response induced by ultrasonic waves or AS. Again, no clinical evidence exists on the immunomodulation in the brains of AD patients or healthy, non-demented individuals under the three types of stimulation reviewed here.

##### TUSS

The reviewed studies present conflicting outcomes concerning the effect of TUSS on the activity of glial cells (i.e., microglia, astrocytes, or macrophages) in the brains of AD transgenic mice. Most of the preclinical TUSS studies have reported activation of immune cells after treatment and their increased co-localization/internalization of Aβ [36, 56–61] or Tau [45] fragments in the brains of AD transgenic models. It is worthy to note that, despite the microglial activation (e.g., extended branching) after ultrasonic treatment, Leinenga and Gotz (2015) observed no alterations in inflammatory markers in the brains of aged APP23 mice (which exhibit pronounced Aβ plaques) after US (once per week, 4 or 7 weeks period) [36]. On the other hand, two studies have reported a reduction in gliosis ( by assessment of glial fibrillary acidic protein [GFAP] [56] and ionized calcium-binding adaptor molecule 1 [Iba1] [56, 57]) in the brains of AD mouse models, either after a single TUSS session of 1 min (marked effect in the hippocampus and entorhinal cortex of 5xFAD mice) [56], or after a repetitive TUSS treatment (3 sessions/20 min each on alternate days, the first week of each month for three months) [57]. Note that the latter study used a significantly higher pulsing rate (6 kHz) and longer treatment duration compared to the others and found a unique regulation of some immune-related genes (e.g., GFAP, Olig2, and CXC chemokine receptor 4) in the brains of 5xFAD mice after US treatment [57].

Moreover, Jordão et al. (2013) have demonstrated that TUSS (single session of 8 min) increases the bioavailability of endogenous antibodies (i.e. immunoglobulins G and M) in the brains of Aβ-based transgenic mice (TgCRND8), and also promotes the interaction of these antibodies with Aβ plaques, thereby facilitating the clearance of their aggregates from the brain [59]. The increased antibody bioavailability is also accompanied by a phagocytic response of glial cells towards Aβ aggregates in both US-treated and non-treated brain hemispheres [59], which is in line with previous reports supporting a US-induced systemic bioeffect in brain areas beyond the one that was stimulated [45]. Moreover, Lee and colleagues (2020) found a marked increase of Aβ peptides in the cerebrospinal fluid space in the TUSS-treated 5xFAD mice, in which ligation of lymphatics to the deep cervical lymph nodes was performed (single session of 1 min) [56]. The data from this study suggest that, although the reactivity of glial cells has been reduced by TUSS, Aβ clearance could be ensured by the lymphatic system, which is possibly activated/stimulated by TUSS.

##### AS

The 40-Hz AS has been shown to modulate the immune response in the brains of AD transgenic mice with intense Aβ plaque burden, by increasing microglial activity [30, 48] and astrocyte reactivity [30] (1 h/day, 7 days period [30]; 2 h/day, 14 days period [48]). However, the one-week-long AS study observed that the increase in microglial activation induced by AS was reversed one week after the end of the treatment [30]. Both studies have reported a decrease in soluble and insoluble Aβ species [30, 48], possibly resulting from microglial activation. As the expression of inflammatory markers was not assessed, it was unknown if this increase in glial activation prompted or regulated neuroinflammation response. Importantly, combination of AS with visual stimulation delivered at 40 Hz (1 h/day, 7 days period) induced microglia to display a unique encapsulating effect around Aβ plaques throughout the brain of 5xFAD mouse, and this effect was not observed for any stimulus alone [30]. Importantly, this microglial activation results in decreased Aβ deposits in the brains of AD transgenic mice [30].

#### Neurotoxicity and neuroplasticity deficits

This section summarizes and discusses the effect of mechanical stimulation on different parameters of neuronal malfunction and neurodegeneration in AD, such as neuronal loss, synaptic malfunction, deficits in neurostructural plasticity (e.g., dendritic atrophy, spine loss), among others. Unfortunately, there is a lack of evidence for WBV or AS from human or animal studies. Therefore, this section only includes animal studies on TUSS.

TUSS treatment of various durations is associated with different beneficial effects against Aβ- and Tau-induced toxicity and structural damage in the brains of AD transgenic mouse models. For instance, Shen (2019) and Lee (2020) led two experiments in which 1-Hz ultrasonic pulses (single session of 1 min) were applied to 3xTg-AD and 5xFAD mice, respectively, which display advanced Aβ and Tau neuropathologies [56, 58]. The authors showed that the ultrasonic waves can modulate the proteomic profile in the brains of AD transgenic mice, which was correlated with enhanced neuronal function [58] and decreased neuronal loss [56] triggered by TUSS. In a study with longer TUSS stimulation, Burgess et al. (2014) found several morphological improvements (i.e., increased number of immature neurons, total dendrite length, and dendrite branching in pre-existing/mature neurons) in the hippocampus of TgCRND8 mice (8 min once a week, 3 weeks period) [38], supporting that the beneficial effect of TUSS was also affecting different parameters of brain plasticity.

Also, Karakatsani et al. (2019) found that the brain slices from treated experimental animals show intact neural network integrity and neuronal morphology after unilateral target TUSS (single session of 1 min), indicating treatment safety [45]. Additionally, Pandit and others (2019) have found TUSS-evoked increased autophagy in neurons (but not in glial cells) in Tau-transgenic K3 mice (2.4 min once a week, 15 weeks period) [66], showing a US-driven improvement of neuronal proteostasis and increased Tau degradation after an extended treatment period. Moreover, Eguchi and colleagues (2018) showed that TUSS in 5xFAD mice (60 min once a week, 12 weeks period) leads to the upregulation of endothelial nitric oxide synthase, neurotrophins, and heat-shock protein 90, accompanied by the downregulation of amyloid precursor protein (APP) and beta secretase-1 (BACE-1), which are associated with a reduction of Aβ species and plaques throughout the brain after TUSS [57]. Importantly, Pandit et al*.* (2019) have also reported reduced p-Tau levels and neurofibrillary tangles in the hippocampus of an AD mouse model, possibly related with the increased autophagy [66]. Again, no clinical evidence has been provided on the mitigation of neurotoxicity after US treatment in humans.

#### Brainwave entrainment and brain functional connectivity

##### WBV

Starting with this therapeutic modality, only one of the reviewed papers has assessed the impact of an eight-week WBV program on neuronal activity of the human brain [67]. Specifically, this study reported increased brain network activity (assessed by electroencephalography, EEG) overtime after 20-Hz to 40-Hz vertical vibration (frequency increased over treatment time) in women with senile dementia [67].

##### TUSS

US treatment has also been proven to increase neuronal activity, despite the limited experimental evidence on the topic. Indeed, among the reviewed studies, only one animal study has reported the occurrence of strong and transient entrainment of 40-Hz oscillations in the brains of 5xFAD mice during TUSS pulsing at the same frequency (single session of 1 h) [61]. At the clinical level, two studies have reported alterations in brain networks after US therapy in AD patients. Meng and colleagues (2019) observed a decreased resting-state functional connectivity in the right frontoparietal networks during BBB opening, although the functional organization was restored at the one-day and one-week follow-ups (2 US sessions, one-month apart) [68]. In the same study, the US-treated patients showed a small tendency of increase of brain connectivity at the 3-month follow-up, both in the right frontoparietal and in the default mode networks [68]. In fact, Beisteiner et al*.* (2020) have studied the effect of 6–12 sessions delivered within the same time window – 1 month period – and detected enhanced memory networks, as well as increased steady-state and task-based functional connectivity in the hippocampus, cortex regions, and the precuneus, and in bilateral hippocampi, respectively [69]. These findings suggest that transcranial US application could be effective in modulating brain activity and networks possibly by adopting frequent sessions over a longer period. In addition, the brain network modulation is correlated with cognitive improvements in AD patients, after both two- or four-week interventions [69]. Thus, it is quite plausible that the periodicity of stimulation can greatly affect the effectiveness and durability of the treatment.

##### AS

Five out of the six reviewed studies assessing the effect of AS (two preclinical and three clinical studies) have shown that AS is effective in increasing neuronal activity [30, 31, 48, 49, 70]. The two preclinical AS studies observed increased gamma power after delivery of 40-Hz intermittent sound waves in 3–8 month-old wild-type (C57BL/6 J) mice (1 h/day, 7 days period) [30], and in 5xFAD transgenic mice (2 h/day, 14 days period) [48]. Interestingly, evoked gamma brain oscillations promoted microglia aggregation in both transgenic mice and their wild-type littermates [48], suggesting that this immunomodulation response is not necessarily activated in response to Aβ and/or Tau pathologies. This brainwave entrainment was accompanied by increased functional connectivity in frontal gamma coupling with parietal delta at the baseline, which swapped to frontal gamma coupling with parietal theta after TUSS [48]. However, an exaggerated increase of evoked gamma brainwaves was observed one day after the intervention, which returned to baseline levels during the rest of the experiment [48], indicating an acute and transient response. In addition, Martorell et al. (2019) have shown that 40-Hz visual stimulation combined with AS delivered at the same frequency (1 h/day, 7 days period) shows a greater gamma oscillation entrainment followed by decreased Aβ load in the brains of 5xFAD mice when compared to each of the stimuli alone.

In line with these findings, the first, and only so far, clinical evidence on the improvement of brain network activity after AS combined with visual stimulation in AD patients is provided by Calomeni and colleagues (2017) (15 min/day on alternate days, 20 days period) [49]. This study found that these sensory stimuli delivered at 8, 10, 12, 14, and 15 Hz (3 min/frequency) increase alpha (i.e. 8–15 Hz) brainwave activity in AD patients, but not in non-demented individuals [49], which is associated with memory gain [49]. Moreover, Papalambros and colleagues performed two experiments in 2017 and 2019, in which they assessed the effect of 20-Hz AS (single session of 4 min delivered during sleep) on the neural activity in healthy non-demented older subjects [70] and patients with amnestic MCI, a precursor form of AD [71]. In the first study performed in healthy non-demented older subjects [70], a unique increase of slow-wave brain activity in the delta range (i.e. 0.5–4 Hz) during stimulation is detected, accompanied by a small tendency of enhanced theta and fast spindle power. Moreover, increased signal amplitude and spindle density are observed during stimulation for all potentials, as assessed by EEG [70]. Importantly, the increased slow-wave activity (i.e. 0.5–4 Hz) is associated with better performance in word recall after stimulation [70]. By applying the same AS protocol to amnestic MCI patients, the authors have found increased slow-oscillations (i.e. 0.5–1 Hz), slow-wave and sigma (i.e. 9–15 Hz) activities during stimulation, whereas no effect was noticed in theta (4–7 Hz) or beta (16–20 Hz) bands [31]. Again, the alterations in neural activity are correlated with improved memory and cognition, as assessed by the cued-recall and verbal paired-associate tests, as well as by the NIH Toolbox Cognition Battery [31].

#### Behavioral performance

The reviewed studies typically present outcomes associated with motor and behavioral abilities. Twenty-seven out of the 37 analyzed papers provided evidence on the effect of mechanical vibration-based stimulation on motor and cognitive performance.

#### WBV

Despite the differences among the protocols (vibrations at 25 ± 15 Hz, 5-mm maximum amplitude, and heterogeneous sessions and treatment durations), studies have shown that the WBV treatment can improve different aspects of physical function, such as balance [21, 72–76], functional mobility [21, 72–79] and muscle mass and/or strength, mainly in the lower limbs [72, 76–78]. Importantly, three studies have reported an enhancement of quality-of-life metrics after WBV training (6–9 weeks period) [21, 73, 78], which are directly associated with motor improvement, as well as with patients’ well-being [80]. In addition, Kim and Lee (2018) showed that WBV could improve cognitive function along with increased brain connectivity and plasticity in senile-demented women undergoing a WBV regimen with increasing frequency (20–40 Hz) of vibrations over time (4 min once a week, 2 months period) [67]. However, the beneficial effect of WBV treatment in non-demented older adults remains unclear, as a WBV treatment of 10 weeks (1–5 min twice a week, 10 weeks period, with increasing training volume over time) described a small tendency (but not significant effect) of functional improvement as assessed by different motor ability tests like walking speed metrics [81]. Future studies should clarify whether the WVB treatment is also beneficial for non-demented older individuals.

##### TUSS

TUSS is also associated with improvement of motor and cognitive skills in AD animal models, including enhanced different types of memory (i.e. recognition, spatial, working, and short- and long-term memories) [36, 56, 58, 66], improved exploratory skills [36, 38, 57] and improved motor ability and coordination [66]. Interestingly, after weekly sessions of 2.4-min delivery over 15 weeks (the longest of the reviewed protocols), no changes in grip strength (a motor-related parameter) were found in K3 mice. [66]. In addition, a study in aged Aβ-positive dogs with advanced Aβ brain pathology found no significant changes in gait, postural reaction or cranial nerve testing after TUSS (2–3 min/ session, once a week, 4 weeks period) [62].

At the clinical level, Lipsman et al. (2018) did not find significant changes in cognition and daily functioning of AD patients at 3-month follow-up after TUSS (7.5 min once a week, 3 months period) [63]. However, in the same study, one of the five treated patients showed a transient cognitive improvement at 1-month follow-up [63]. Similarly, other studies have detected no significant changes in cognitive function in AD patients after two to five sessions of continuous ultrasonic waves (220-kHz center frequency) (2 weeks period), either at 30-day [82] or at 1-week follow-up [65], independent of alterations in Aβ burden. On the other hand, one study has demonstrated that a US treatment delivered in 5-Hz ultrasonic pulses (2 to 4 weeks period) is able to improve cognitive performance (e.g. enhanced memory and verbal processing) in a standardized neuropathological assessment defined at the Consortium to Establish a Registry for Alzheimer's disease (CERAD), in a cohort of AD patients right after the treatment as well as three months later [69]. Importantly, the cognitive improvement of these AD patients is correlated with increased functional connectivity between different brain areas (e.g. hippocampus, parahippocampal cortex, parietal cortex) [69].

##### AS

For AS, a majority of evidence for its beneficial impact on behavioral performance has arisen from clinical rather than animal studies. Only the animal study by Martorell et al. (2019) reported improved spatial memory and recognition in 5x-FAD mice after 40-Hz AS (1 h/day, 7 days period), as assessed by different behavioral tests (i.e., Morris water maze, novel object recognition, and novel object location tasks) [30]. In human studies, multiple insights on behavioral performance and cognitive function have been recently published. For instance, Papalambros et al*.* assessed the impact of a 20-Hz AS intervention (single session of 4 min during sleep) in non-demented elderly [70] and amnestic MCI patients [31] and found a positive effect on morning word-recall in MCI patients [31].

Finally, vibroacoustic stimulation (type of AS-) combined with visual stimuli, both delivered at 40 Hz (30 min twice a week, 6 weeks period), improves cognitive performance after stimulation in AD patients, as well as the awareness of surroundings, the interaction and discussion/storytelling abilities, and alertness [83]. Overall, the data suggest that 40-Hz stimulation delivered in different rhythmic sensory-based modalities is a promising tool to improve different aspects of behavior.

## Discussion

**Fig. 2:**
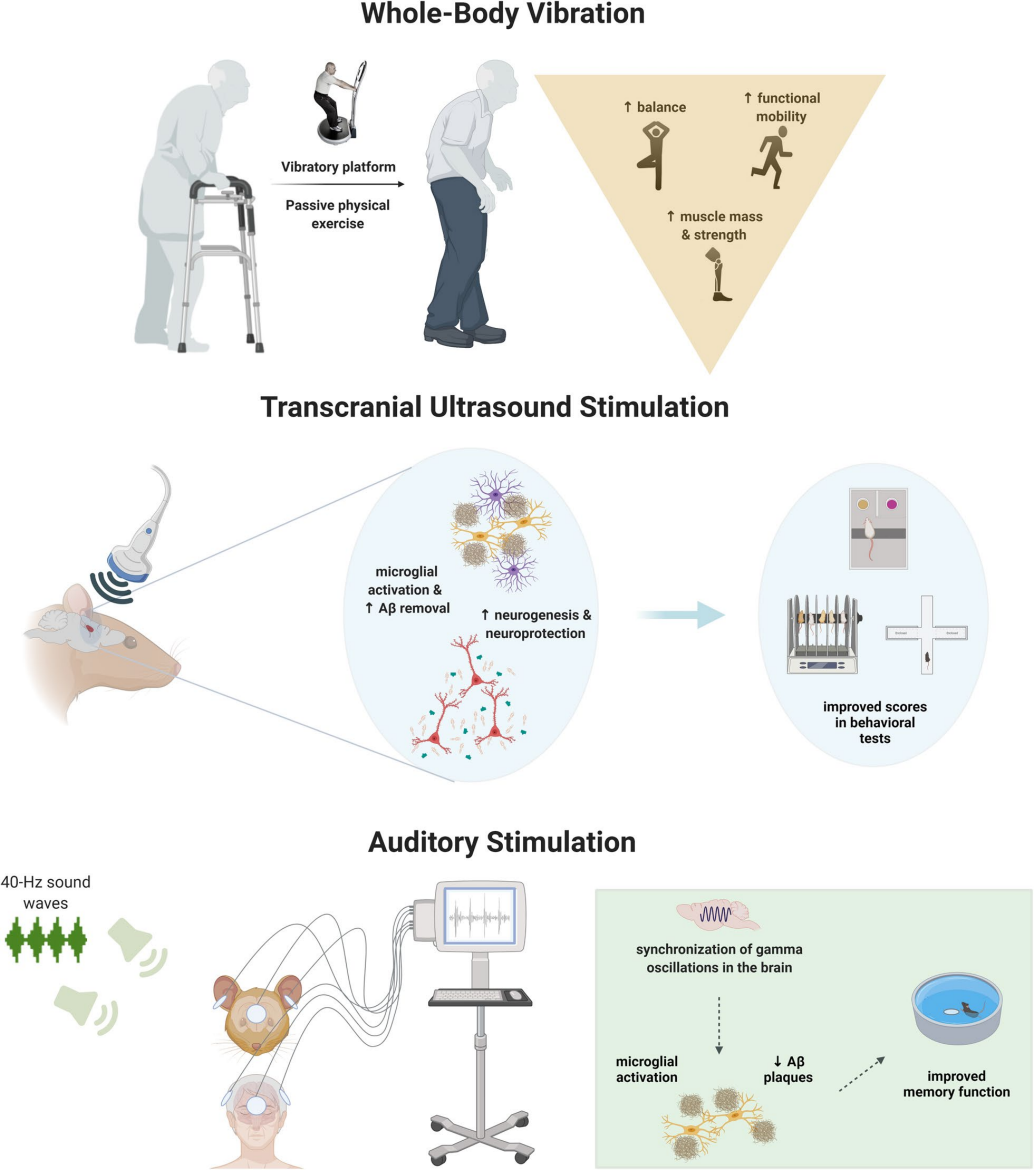
Schematic representation of the main mechanical vibration effects on AD pathology and age-related impairments. Created with BioRender.com

The ability of noninvasive, mechanical vibration strategies to impact AD pathology and related behavioral deficits has received increasing attention over the last years. Hereby, we provide a systematic review comparing and discussing cellular, molecular, functional, and behavioral outputs of three mechanical-based stimulation modalities and the most promising protocols against the AD (Fig. 2).

### Safety and feasibility of mechanical vibration-based interventions

For the clinical implementation of any therapeutic modality, it is imperative to assess the safety and feasibility of the treatment. Particularly, WBV training requires a wise consideration of such parameters, especially in older individuals. These features have been extensively considered in the reviewed studies, as many of them are important for the participation of the elderly in the implemented programs, including the attendance rate, compliance, feedback provided by the participants, and adverse events. All the reviewed WBV protocols present a high attendance rate (> 74%) and treatment compliance (> 73%), and scarce adverse effects (e.g., muscle pain and discomfort) are reported after vibratory stimulation. The implemented protocols are well-tolerated by the majority of the participants. For instance, while a 30-Hz WBV training protocol in demented patients is reported as enjoyable by the participants [84], another study of 26-Hz vibrations reported that the subjects felt high discomfort, forcing the protocol to be adapted to a vibration frequency of 18 Hz; yet, the 18-Hz protocol was also poorly tolerated by the elderly [81]. These data suggest that the discomfort is not frequency-dependent, but possibly caused by a greater amplitude of vibration. Indeed, WBV protocols with vibration rates over 30 Hz have proved safe and well-tolerated by the elderly among the reviewed studies [21, 77, 79, 84]. In contrast, high-amplitude vibration should be avoided to prevent distress and dropouts from the WBV training. Importantly, the reviewed studies suggest that WBV protocols are suitable even for frail patients [76, 78, 81] and individuals with cognitive impairments [21, 67, 84], as they do not require any physical effort or ability and can also be personalized for each patient according to his anatomical and physiological features. Taken together, these data demonstrate that WBV should be added to medical and rehabilitation therapies in older patients, enabling the enhancement of their neuromuscular performance and, possibly, cognitive skills.

Regarding the US modalities, the US-mediated BBB opening has proved safe, transient, and effective in all the reviewed protocols. No major adverse events occur and TUSS is well tolerated by all animals and patients. However, among the studies reviewed, no long-term effects were assessed, with the longest follow-up period in AD patients being three months [69]. Nevertheless, a study in healthy non-human primates employing repeated ultrasonic waves over four to 20 months has reported no damage in the animal brain, and no alterations in health status (e.g., respiration rate, blood pressure, and O_2_ level) throughout the intervention [85]. These findings support the long-term safety of US treatment in primates, but this issue needs to be addressed in future transcranial US studies in both demented and non-demented patients, mainly after multiple-sonication protocols. Importantly, to prevent tissue damage and ensure appropriate sonication power, some of the reviewed studies [38, 63, 68] used an acoustic feedback controller algorithm proposed by O’Reilly and Hynynen (2012) [86] that allows the real-time monitoring of acoustic emissions. The system detects subharmonic emissions that indicate enhanced microbubble activity, and responds by reducing the applied acoustic pressure in the stimulation site. This enables the control of ultrasound emission according to microbubble response, avoiding in situ pressure fluctuations due to skull thickness or vasculature variations among individuals [38]. This represents an important development in the field in terms of clinical translation of TUSS for AD, contributing to good tolerability and safety.

Finally, sparse information exists on the safety and feasibility of AS protocols since this sensory modality does not require any effort, or induce any pain or discomfort in participants. In fact, when applied overnight, both healthy non-demented older adults and amnestic MCI patients report no changes in sleep quality and sleep-staging features [31, 70], suggesting that AS is comfortable and suitable even for demented elderly persons.

### Most relevant outcomes of multi-mechanical stimulation

Based on the reviewed studies (Table 5), we found that the main outcomes associated with the WBV program in the elderly are improvement of different aspects of motor performance, such as mobility and balance skills, as well as increased muscle mass and/or strength. The majority of the reviewed studies have reported enhancement of at least one of these parameters, although a wide variety of WBV protocols have been implemented. Intriguingly, we noticed some divergent outcomes even for similar intervention parameters. We assume that this divergency is associated with differences in the patient’s physical/motor status at baseline. As suggested by Lam (2017), worse baseline physical/motor status allows for a greater gain of physical and/or cognitive performance after WBV treatment [21], which can explain the lack of significant results in some studies due to insufficient mobility impairment, and so, less improvement. Although this approach seems to be more efficient in people with worse neuromuscular function, this does not mean that non-frail subjects cannot benefit from WBV training. In fact, many studies have reported marked improvement of motor and cognitive performances in young and healthy adults [87–90]. Future studies in older individuals should address the baseline functional mobility and cognitive status to understand the suggested reversed relationship between improvement and health status.

Nevertheless, it has been demonstrated that the implementation of WBV training programs in demented and cognitively impaired individuals is effective in improving the overall neuromuscular function, functional mobility, and metrics related to the quality of life [21, 73, 78]. Simultaneously, this training may also improve disrupted brain networks and many aspects of cognitive function, such as orientation, memory, and linguistic skills [67]. Importantly, by enhancing the overall motor ability, WBV exercise may constitute an effective strategy to prevent falls and therefore bone fractures [76, 91], as well as brain damage due to trauma, which are common in advanced age [92, 93]. Furthermore, the improved neuronal activity and cognitive enhancement induced by WBV in senile-demented women [67] point towards further investigation on the effect of WBV on cognitive status and brain pathology in AD or demented patients.

On the other hand, TUSS can provide noninvasive immunoregulation and neuromodulation reversing AD pathomechanisms. Glial cells play a critical role in maintaining the balance in the nervous system, mainly  when something disrupts its functioning. For instance, in the AD brain, microglia and astrocytes play an essential role in controlling the levels of aggregated proteins (mainly Aβ), promoting their clearance. For this reason, many researchers have focused on immunotherapies to increased Aβ clearance through administration of immunotherapeutic agents, both related and non-related with microglial activation [94], although non-satisfactory results have been obtained in clinical trials [95]. Here, many of the TUSS studies have shown activation of different immune cells, enhanced co-localization with Aβ and Tau followed by reduced Aβ or Tau accumulation in the brains of different AD transgenic mouse models [38, 66]. In fact, several researchers have reported enhanced co-localization of microglia around Aβ or Tau aggregates in the brains of AD transgenic mice after US stimulation [36, 45, 56–61] as well as 40-Hz AS combined with visual stimulus in 5xFAD mice [30]. Importantly, these alterations are accompanied by a reduction of Aβ or Tau deposits [45, 56–59, 61]. Stimulated by Iaccarino et al*.* (2016) who used 40-Hz visual stimulation to produce gamma brainwave entrainment in the brains of AD transgenic mice, many research efforts have provided strong evidence about the effectiveness of TUSS pulsing at low frequency. For instance, 5-Hz and 40-Hz USS modulate brain network activity in AD transgenic mice [61] and patients [69], respectively. Moreover, pulsed TUSS evoked neurogenesis and dendritic growth in the hippocampus of TgCRND8 mice [38, 57]. This evidence supports the growing interest in ultrasonic wave-based treatments to fight against AD brain pathology, not only by blocking or reversing Aβ and/or Tau pathology but also by attenuating inflammation, neuronal and synaptic loss, and altered brain activity.

As AD patients present abnormalities in gamma oscillations that are essential for high-order cognitive functions, such as information storage and retrieval [54], these brainwaves have been used as a target in emerging strategies to address altered brain networks in the AD brain. The sensory-based modalities delivered as rhythmic stimuli, which particularly focus on the gamma frequency band, have gained major attention in the last five years to improve altered brain oscillations; increased brainwave power and synchronization seem to affect Aβ and/or Tau pathologies [30, 48, 51, 54]. In fact, the association between brainwave entrainment and AD neuropathology has been widely studied, as researchers have found that the entrained brain oscillations can modulate AD neuropathology, reducing Aβ and/or Tau pathology [51, 54, 96]. Moreover, other frequency bands have also been associated with memory and cognitive enhancements. For instance, visual and auditory stimuli delivered at alpha and beta frequency bands can increase brain oscillations in EEG signals associated with memory enhancement [49]. Interestingly, these stimuli affect brainwave activity differently according to the conditions of the participants, as the applied therapy increases alpha activity in the brains of AD patients while no significant effect was produced in patients with Parkinson’s disease and non-demented older individuals [49]. This suggests that the applied protocol is more effective in improving neuronal network dysfunctions associated with AD neuropathology. Similarly, Papalambros et al*.* (2017 and 2019) observed increased slow-wave activity (i.e. 0.5–4 Hz) in both non-demented and demented patients after 20-Hz AS (beta frequency band) overnight, which is correlated with cognitive enhancement [31, 70]. Interestingly, these studies show that the increased power of specific brainwaves is not necessarily associated with stimuli delivered in the same frequency band, suggesting that rhythmic AS can increase brain activity across different frequency bands.

### Most promising protocols for clinical application

In WBV studies, the passive exercise training is delivered using two distinct vibration modes, namely vertical and rotational vibration. These modes differ in terms of vibration mechanisms, the direction of vibration, and stimulated muscles, and hence they can produce different outcomes in similar cohorts. For this reason, it is important to clarify which aspects of these vibration modes can lead to beneficial or detrimental effects in the frail elderly. On the one hand, mechanical acceleration in rotational vibration is performed in the anterior–posterior axis [14], which is coincident with the direction in which the lower limb balance declines over the aging process; rotational vibration can also cause muscle and joint pain [97]. However, rotational vibration has been proven to promote an increase in muscle strength, which has been associated with the increased balance and functional mobility [77, 98]. On the other hand, in the WBV training with vertical vibration, the platform moves up and down, which mobilizes all body weight, toning muscles and strengthening bones [99]. As both types of vibration are associated with positive outcomes, further investigations should compare and assess the impact of different directions of the vibration in the elderly and particularly AD patients. In addition, many studies have used a WBV protocol with progressively increased frequency of vibration over time, which is associated with marked improvements in neuromuscular performance and/or quality of life [73, 77–79]. This suggests that increasing the training intensity during the WBV treatment may elicit more beneficial effects than using a constant vibration frequency. In summary, data extracted from the reviewed studies suggest that the main parameters to be employed in future AD clinical research on WBV protocols are: (i) vertical vibration, as only this type of vibration is used for cognitively impaired subjects; (ii) pulse frequency of 20–40 Hz, with special emphasis on 30-Hz vibration and increased frequency over time; (iii) amplitude of vibration of 2 mm; and (iv) a training volume of 4–6 min per session, two to four sessions per week over five/six consecutive weeks. Importantly, although the treatment parameters among cohorts are quite similar, there is a tendency for shorter treatment (i.e., lower duration) employed in demented patients compared with non-demented subjects.

Considering TUSS in patients with AD or dementia, the effectiveness needs to be preceded by a demonstration of the intervention safety, mainly due to the potential thermal and damaging effects produced by the US when applied at a high frequency. For this reason, and given the information provided in Table 3, we believe that future protocols designed to affect AD pathology should consider: (i) the frequency of the pulse, as most of the reviewed preclinical studies used intermittent stimulation (commonly from 1 to 10 Hz [36, 37, 58, 66, 69], although 40-Hz TUSS was shown to produce strong but transient signal entrainment in the brains of AD transgenic mice [61]); (ii) the delivery mode, as the whole-brain stimulation (e.g. through a helmet-like device) seems to be the most appropriate option to produce a widespread effect over the brain, although localized sonication has proved to affect further brain regions in mice [45, 56, 59]; and (iii) the number of sonications, as a minimum of six sessions are required.

Finally, concerning the application of AS in AD and cognitively impaired patients, the most important parameters for the effect of AS on AD pathology are: (i) pulsing rate of 40 Hz, which represents the most promising option (essentially based on AD transgenic mice studies); and (ii) stimulation volume of 15- to 30-min sessions, at least once per week, over a minimum of three weeks. However, a single session of overnight AS during sleep is also effective in increasing slow-wave activity and enhancing memory recall in non-demented older adults [70] and in amnestic MCI [31], which means that AS delivery during sleep should be further investigated in AD patients in future trials.

Overall, the reviewed protocols show that various combinations of protocol specifications can be employed to improve different aspects of AD pathology and to improve cognition and other behavioral aspects. Currently, quite similar treatment parameters have been employed in both demented and non-demented subjects. In further studies in AD and demented patients, it is necessary to determine which protocol specifications should be employed to impact AD pathology.

### Limitations and future directions

The main limitation associated with the reviewed studies is the lack of histological/biochemical and/or imaging assessment of key biomarkers, such as Aβ and Tau species, and neuronal damage/inflammation markers in the brains of participants. This monitoring would enable further advances in the development of mechanical-based treatments to block AD pathomechanisms. Most of the studies performed in humans essentially focus on brain network activity and motor and behavioral improvements, while only three studies have monitored alterations at biochemical and functional/structural levels (e.g., serological analysis to assess biomarkers, and brain imaging techniques such as PET and MRI) after mechanical wave treatment [63, 65, 100]. Therefore, additional studies that use mechanical-based stimulation modalities and report biochemical and imaging brain data are needed to clearly describe the bioeffects of mechanical-based stimulation modalities on different molecular and metabolic aspects of AD neuropathology.

Moreover, longer follow-up periods are needed to test the effect of detraining or treatment suspension, as most of the reviewed studies with a follow-up period have reported a decline/reverse of the positive effect right after the treatment [37, 59, 63, 76]. In fact, no clinical data have been published so far on the long-term effect of these stimulation modalities, so a longer study span is needed in the future.

In addition, many of the reviewed studies present a lack of important information related to protocol specifications, e.g. the amplitude of vibration [67, 75] and the type of vibration [84] in WBV protocols, as well as the US center frequency [69] and pulsing rate [63, 65, 82] in TUSS studies. Other parameters such as the contact area and pressure of transducer on the heads of animals/patients, the distance and angle between the transducer and the head, the duty cycle, and the intensity of acoustic signal (e.g., power density) are required to fully characterize the mechanical stimulation protocol, which allows for reproducibility in experiments and a reliable comparison among different studies.

## Conclusions

Latest studies have reported the effectiveness, safety, and feasibility of mechanical waves delivered through different stimulation modes to mitigate AD pathology and to improve the overall physical and behavioral performance of AD patients, demented individuals, and non-demented elderly persons. Importantly, clinical evidence from AD and demented patients has accumulated over the past five years, and promising results have been generated. Based on the current evidence, the low-cost and noninvasive mechanical-based interventions can contribute to the development of an alternative effective solution to fight AD neuropathology and related behavioral deficits. However, there is limited evidence on the comparison between treatment specifications, and hence, suboptimal and quite distinct protocols have been employed. This comparison is crucial to further optimize the stimulation protocols in order to maximize their therapeutic potential. Nevertheless, the effectiveness of the mechanical-based stimulation strategies in promoting physiological and electrochemical alterations that could mitigate AD pathology will certainly evolve in the future and, hopefully, revolutionize AD therapeutics in the upcoming decades.

## Supplementary Information

**Additional file 1.Table S1.** Quality assessment data for clinical trials, using the EPHPP Quality Assessment Tool. **Table S2.** Quality assessment data for animal studies, using the STAIR preclinical recommendations.

## Data Availability

Not applicable.

## Abbreviations

Aβ: Amyloid-β
AD: Alzheimer’s disease
APP: Amyloid precursor protein
AS: Auditory stimulation
BACE-1: Beta secretase-1
BBB: Blood–brain barrier
EEG: Electroencephalography
EPHPP: Effective Public Health Practice Project
MCI: Mild cognitive impairment
PET: Positron emission tomography
PRISMA: Preferred Reporting Items for Systematic Reviews and Meta-Analyses
STAIR: Stroke Therapy Academic Industry Roundtable
TUSS: Transcranial ultrasound stimulation
US: Ultrasound
WBV: Whole-body vibration
