# Supplementary material for: Multi-mechanical waves against Alzheimer’s disease pathology: a systematic review
Source: Transl Neurodegener. 2021 Sep 24;10:36. doi: 10.1186/s40035-021-00256-z (PMC8464104; doi:10.1186/s40035-021-00256-z)
Supplement: Supplementary file 1 — Additional file 1.Table S1. Quality assessment data for clinical trials, using the EPHPP Quality Assessment Tool. Table S2. Quality assessment data for animal studies, using the STAIR preclinical recommendations. [file 40035_2021_256_MOESM1_ESM.docx]

**Table S1.** Quality assessment data for clinical trials, using the EPHPP Quality Assessment Tool.

|  | **Selection bias** | **Study design** | **Confounders** | **Blinding** | **Data collection methods** | **Withdrawals and drop-outs** | **Final decision** |
| --- | --- | --- | --- | --- | --- | --- | --- |
| **Whole-body vibration** |  |  |  |  |  |  |  |
| Kawanable *et al.* (2007) [1] | Weak | Weak | Strong | Moderate | Strong | Weak | **Weak** |
| Cheung *et al.* (2007) [2] | Strong | Strong | Strong | Moderate | Strong | Strong | **Strong** |
| Furness and Maschette (2009) [3] | Strong | Strong | Weak | Moderate | Strong | Strong | **Moderate** |
| Cardinale *et al.* (2010) [4] | Strong | Strong | Strong | Moderate | Strong | NA | **Strong** |
| Machado *et al.* (2010) [5] | Strong | Strong | Strong | Moderate | Strong | Strong | **Strong** |
| Marín *et al.*  (2011) [6] | Strong | Strong | Strong | Moderate | Strong | Strong | **Strong** |
| Beaudart *et al.* (2013) [7] | Strong | Strong | Weak | Moderate | Strong | Strong | **Moderate** |
| Zhang *et al.* (2014) [8] | Strong | Strong | Strong | Moderate | Strong | Strong | **Strong** |
| Sievänen *et al*. (2014) [9] | Strong | Strong | Strong | Moderate | Strong | Strong | **Strong** |
| Álvarez-Barbosa *et al.* (2014) [10] | Strong | Strong | Strong | Moderate | Strong | Moderate | **Strong** |
| Lam *et al.* (2017) [11] | Strong | Strong | Strong | Moderate | Strong | Strong | **Strong** |
| Kim & Lee (2018) [12] | Strong | Weak | Strong | Moderate | Strong | Strong | **Moderate** |
| Zhu *et al.* (2019) [13] | Strong | Strong | Strong | Moderate | Strong | Strong | **Strong** |
| Heesterbeek *et al.* (2019) [14] | Strong | Strong | Strong | Moderate | Strong | Strong | **Strong** |
| **Transcranial ultrasound stimulation** |  |  |  |  |  |  |  |
| Lipsman *et al.* (2018) [15] | Strong | Moderate | Strong | Moderate | Strong | Strong | **Strong** |
| Meng *et al.* (2019) [16] | Strong | Moderate | Strong | Moderate | Strong | Strong | **Strong** |
| Beisteiner *et al. (*2020) [17] | Strong | Moderate | Strong | Moderate | Strong | Strong | **Strong** |
| Rezai *et al.* (2020) [18] | Moderate | Moderate | Strong | Moderate | Weak | Strong | **Weak** |
| D’Haese *et al.* (2020) [19] | Moderate | Moderate | Strong | Moderate | Moderate | Strong | **Moderate** |
| **Auditory Stimulation** |  |  |  |  |  |  |  |
| Clements-Cortes *et al.* (2016) [20] | Strong | Strong | Strong | Strong | Strong | Strong | **Strong** |
| Calomeni *et al.* (2017) [21] | Strong | Moderate | Strong | Moderate | Strong | Strong | **Strong** |
| Papalambros *et al.* (2017) [22] | Strong | Strong | Strong | Moderate | Strong | Strong | **Strong** |
| Papalambros *et al.* (2019) [23] | Strong | Strong | Strong | Moderate | Strong | Strong | **Strong** |

NA – not applicable.

**Table S2.** Quality assessment data for animal studies, using the STAIR preclinical recommendations.

| **Checklist**  **Reference** | **Sample size calculation** | **Inclusion and exclusion criteria** | **Randomization** | **Allocation concealment** | **Reporting of animals excluded from analysis** | **Blinded assessment of outcome** | **Reporting potential conflicts of interest and study funding** |
| --- | --- | --- | --- | --- | --- | --- | --- |
| **Transcranial ultrasound stimulation** |  |  |  |  |  |  |  |
| Jordão *et al.* (2013) [24] | Weak | Strong | Moderate | Weak | NA | Weak | Strong |
| Burgess *et al.* (2014) [25] | Weak | Strong | Moderate | Weak | Strong | Weak | Strong |
| Leinenga & Götz (2015) [26] | Weak | Strong | Strong | Strong | Weak | Strong | Strong |
| O’Reilly *et al.* (2017) [28] | Weak | Strong | Weak | NA | NA | Moderate | Strong |
| Leinenga & Götz (2018) [29] | Weak | NA | Moderate | Weak | NA | Strong | Strong |
| Eguchi *et al.* (2018) [30] | Weak | NA | Weak | NA | NA | Strong | Moderate |
| Poon *et al.* (2018) [31] | Weak | Strong | Strong | Moderate | Strong | Strong | Strong |
| Pandit *et al.* (2019) [32] | Weak | Strong | Moderate | Weak | NA | Moderate | Strong |
| Karakatsani *et al.* (2019) [33] | Weak | NA | Moderate | Weak | Weak | Weak | Strong |
| Bobola *et al.* (2020) [36] | Weak | Strong | Weak | NA | NA | Weak | Strong |
| Shen *et al.* (2020) [37] | Weak | Strong | Moderate | Moderate | NA | Moderate | Strong |
| Lee *et al.* (2020) [38] | Weak | Strong | Weak | NA | NA | Weak | Strong |
| **Auditory Stimulation** |  |  |  |  |  |  |  |
| Lee *et al.* (2018) [39] | Weak | NA | Moderate | Weak | NA | Weak | Weak |
| Martorell *et al.* (2019) [40] | Weak | NA | Moderate | Weak | Moderate | Strong | Strong |

NA – not applicable.
